# Differential Seroprevalence of Human Bocavirus Species 1-4 in Beijing, China

**DOI:** 10.1371/journal.pone.0039644

**Published:** 2012-06-22

**Authors:** Li Guo, Yaying Wang, Hongli Zhou, Chao Wu, Jingdong Song, Jianguo Li, Gláucia Paranhos-Baccalà, Guy Vernet, Jianwei Wang, Tao Hung

**Affiliations:** 1 MOH Key Laboratory of Systems Biology of Pathogens and Christophe Mérieux Laboratory, IPB, CAMS-Fondation Mérieux, Institute of Pathogen Biology (IPB), Chinese Academy of Medical Sciences (CAMS) & Peking Union Medical College (PUMC), Beijing, People’s Republic of China; 2 National Institute for Viral Disease Control and Prevention, Chinese Center for Disease control and Prevention, Beijing, People’s Republic of China; 3 Fondation Mérieux, Lyon, France; University of Kansas Medical Center, United States of America

## Abstract

**Background:**

Four species of human bocaviruses (HBoV1-4) have been identified based on phylogenetic analysis since its first report in 2005. HBoV1 has been associated with respiratory disease, whereas HBoV2-4 are mainly detected in enteric infections. Although the prevalence of HBoVs in humans has been studied in some regions, it has not been well addressed globally.

**Methodology/Principal Findings:**

Cross-reactivity of anti-VP2 antibodies was detected between HBoV1, 2, 3, and 4 in mouse and human serum. The prevalence of specific anti-VP2 IgG antibodies against HBoV1-4 was determined in different age groups of healthy individuals aged 0-70 years old in Beijing, China, using a competition ELISA assay based on virus-like particles of HBoV1-4. The seroprevalence of HBoV1-4 was 50%, 36.9%, 28.7%, and 0.8%, respectively, in children aged 0-14 years (n = 244); whereas the seroprevalence of HBoV1-4 was 66.9%, 49.3%, 38.7% and 1.4%, respectively, in healthy adults (≥15 years old; n = 142). The seropositive rate of HBoV1 was higher than that of HBoV2, HBoV3, and HBoV4 in individuals older than 0.5 years. Furthermore, IgG seroconversion of HBoV1 (10/31, 32.3%), HBoV2 (8/31, 25.8%), and HBoV3 (2/31, 6.5%) was found in paired sera collected from children with respiratory tract infections who were positive for HBoV1 according to PCR analysis.

**Conclusions/Significance:**

Our data indicate that HBoV1 is more prevalent than HBoV2, HBoV3, and HBoV4 in the population we sampled in Beijing, China, suggesting that HBoV species may play differential roles in disease.

## Introduction

Human bocavirus (HBoV), a member of the *Parvoviridae* family, is a potential etiologic agent of respiratory disease and of acute gastroenteritis [1]–[11]. Based on phylogenetic analysis of viral genomes, four species of HBoVs (HBoV1-4) have been identified [1], [7]–[9]. HBoV1 is associated with respiratory tract diseases [1], [2], [12]–[14]. HBoV2 and 3 have been detected in the respiratory tract, but are associated mainly with stool samples [8]–[11], [15], [16]. HBoV4 has been detected in enteric infections [9]. However, as HBoVs are frequently co-detected with other viral infections in patients with respiratory or enteric infections, the exact roles of HBoVs in pathogenicity are unclear.

HBoVs are small, non-enveloped viruses with a linear single-stranded DNA genome of approximately 5 kb in length. The genome consists of four open reading frames (ORFs), encoding two nonstructural proteins (NS1 and NP1) and two overlapping capsid proteins (VP1 and VP2) [1]. The lack of a well-established cell culture system or animal model to propagate HBoVs has hampered understanding of the infection and pathogenicity of HBoVs. Studies have shown that the VP2 protein harbors the major antigen of HBoV and can form the empty virus-like particles (VLPs) which mimic HBoV virions morphologically and antigenically. The VP2 VLPs have been successfully used as antigens for detecting antibodies against HBoVs [17]–[22].

Currently, detection of HBoVs nucleic acid is primarily used to estimate the prevalence of HBoV species in clinical samples. The prevalence of HBoV1, which is mainly detectable in children under two years old [23], is 2–19% in patients suffering from acute respiratory tract infections (ARTIs) worldwide as detected by PCR analysis [1], [2], [10], [12], [13], [23], [24]. The detection rate of HBoV2, HBoV3, and HBoV4 DNA in stool samples have been reported as 1-26%, 0.4-5%, and 0-2%, respectively [8], [9], [23], [25]. The prevalence of HBoV2-4 is higher in children than in adults according to some, but not all studies [8], [9], [11], [16], [25]. However, the data collected from patients may not represent HBoV infection in the general population as subclinical infections can occur and HBoV persists in the nasopharynx [26]–[29].

Seroepidemilogical investigations of healthy populations may be more useful than patient studies in assessing the prevalence, spread, and exposure distribution of HBoVs in the population. The seroprevalence data also allow a comparison between the frequency of natural infection and the frequency of this virus in individuals with infections [30]. However, previous seroepidemiological studies of HBoVs have mainly focused on HBoV1. HBoV1 specific IgG antibodies were frequently detected in children, with a seropositive rate ranging from 40.7%-60% for children < four years old and up to >85% for those ≥ four years old [19]. The seropositive rate of HBoV1 VP2-specific IgG antibodies is about 94% in healthy adults [18]. However, Kantola et al. recently showed cross-reactivity between the VP2 VLPs of HBoV1-4, which can largely affect the seropositive data of HBoV species. They showed that after depletion of cross-reactive antibodies, the approximate seroprevalences of HBoV1-4 in adults were 59%, 34%, 15%, and 2%, respectively [22]. However, the samples used in that study were only collected from healthy young people with a narrow range of age, including 115 subjects aged 21-32 years from Finland and 80 subjects aged 18-20 years from Pakistan. Given that the prevalence of a virus infection may vary by age as well as geographically, there remains to be a need to estimate the seroprevalence of HBoVs based on the detection of antibodies against HBoV1-4 from data of a more complete age range and from other global, geographic regions.

In the present study, we used a competition ELISA (cELISA) assay to estimate the seroprevalence of HBoV1-4 in healthy Chinese individuals ranging in age from 0 to 70 years in Beijing, China. We also compared the seroconversion of anti-HBoV IgG antibodies in 31 paired serum samples from ARTI children who were positive for HBoV1 by PCR. Our findings provide informative data for evaluating the prevalence and pathologic roles of HBoVs.

## Results

### Production of HBoV1-4 VLPs

**Figure 1:**
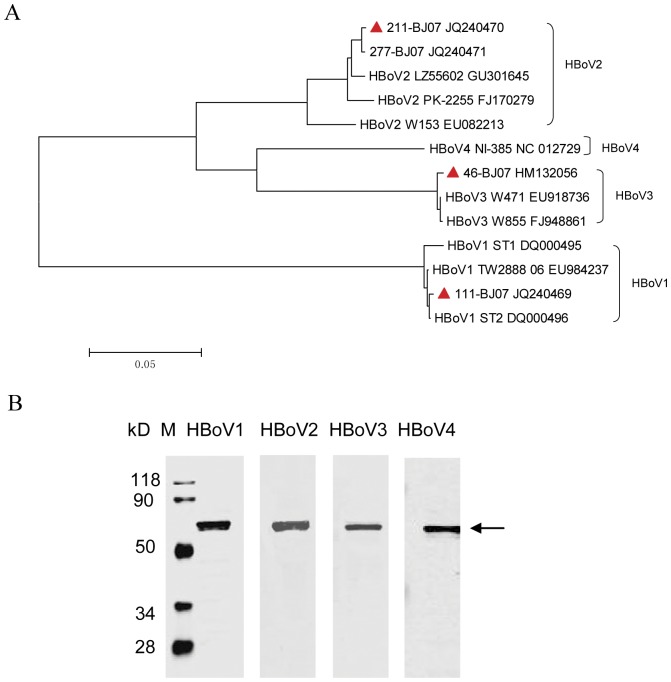
Production of virus-like particles of human bocaviruses. (A) Phylogenetic analysis of human bocavirus (HBoV) species. The phylogenetic tree with 1,000 bootstrap replicates was generated using the Clustal W and MegAlign programs in the MEGA 4.0 software package and based on the complete sequences of the VP2 gene of the HBoV strains used in this study. The VP2 genes of the HBoV1, 2, and 3 were obtained from stool samples from children with acute diarrhea in Beijing and are marked with red triangles. HBoV1 strains ST1, ST2, and TW2888_06; HBoV2 strains W153, PK-2255, LZ55602, and 277-BJ07; HBoV3 strains W471 and W855; and HBoV4 strains NI-385 were used as reference sequences (GenBank accession numbers DQ000495, DQ000496, EU984237, EU082213, FJ170279, GU301645, JQ240471, EU918736, FJ948861, NC012729, respectively). (B) Western blot analysis of the HBoV1-4 VLPs detected by mouse antisera against HBoV1, 2, 3, and 4 VP2, respectively.

To produce antigens that can be used to evaluate the seroprevalence of HBoVs, the VP2 genes of HBoV1-4 were expressed in baculovirus to generate VLPs. The VP2 genes of HBoV1, HBoV2, and HBoV3 were amplified from stool samples by PCR. The sequences of these genes were verified by phylogenetic analysis (Figure 1A). The VP2 genes from respiratory and stool specimens were very similar in sequences. The HBoV1 strain 111-BJ07 used in this study clustered with the reference HBoV1 strain ST2 [1], with homologies of 99.8%. The HBoV2 strain 211-BJ07 has 97.9% identity with the reference HBoV2 strain PK-2255 [7]. The HBoV3 strain 46-BJ07 has 99.6% identity with reference HBoV3 strain W471 [8]. As we did not find any HBoV4 positive samples, the VP2 gene of HBoV4 was synthesized according to the sequence of HBoV4 strain NI-385 [9].

The VP2 proteins of HBoV1-4 were expressed in insect cells using a baculovirus expression system. The generation of HBoV VLPs was verified by ultracentrifuge-purification and electron microscopy (EM). Typical parvovirus-like particles of 22–24 nm in size, similar to the particles of infectious parvovirus virions, were visualized under EM (data not shown). The purified VLPs of HBoV1, 2, 3 and 4 were also reactive to murine antisera specific for the VP2 protein of the respective HBoV species in Western blot assays (Figure 1B).

### Cross-reactivity of HBoVs

Sequence alignment showed a high amino acid identity of VP2 between HBoV species, with 77.5% identity between HBoV1 and HBoV2, 77.7% identity between HBoV1 and HBoV3, 77.5% identity between HBoV1 and HBoV4, 89.4% identity between HBoV2 and HBoV3, 88.5% identity between HBoV2 and HBoV4, and 90.9% identity between HBoV3 and HBoV4 (data not shown), indicating possible cross-reactivity between HBoV species. To evaluate the potential cross-reactivities, we examined the reactivity between the HBoV1-4 VLPs and purified mouse antisera against HBoV1, 2, 3, and 4 VP2 using Western blot and ELISA assays.

**Figure 2:**
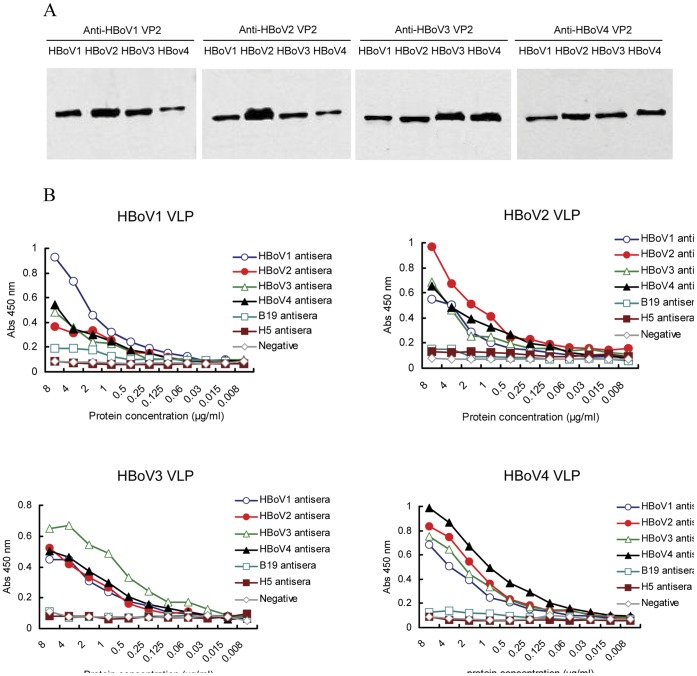
Cross-reactivity between antisera against VP2 proteins of HBoV1, 2, 3, or 4 with virus-like particles of each HBoV species. (A) Western blot analysis. Mouse antiserum against HBoV1, 2, 3, or 4 was diluted at 1∶1000 and incubated with the virus-like particles of HBoV1, 2, 3, and 4, respectively. The loading amount of VLPs for each lane was 400 ng. (B) ELISA assay. Mouse antisera against HBoV1, 2, 3, or 4 were tested for reactivity to virus-like particles of HBoV1, 2, 3, and 4, respectively. Rabbit antiserum against human parvovirus B19 VP2 and mouse antiserum against influenza virus H5 hemaglutinin (HA) were used as controls. The Abs 450 nm values for each concentration of coating antigens are shown on the y-axis; the protein concentrations of antigens coated in ELISA assay are shown on the x-axis.

Western blot analysis showed that the antisera against HBoV1, 2, 3, and 4 reacted with 400 ng of VLPs of each HBoV species, as indicated by detection of specific bands of 60 kD in size (Figure 2A). Similar cross-reactivities were also detected by ELISA assays (Figure 2B). Mouse sera against HBoV1, 2, 3, and 4 reacted strongly with the homologous VLPs. Moreover, all four antisera reacted with the heterologous HBoV VLPs when the concentration of the mice antiserum was high (>0.25 µg/mL). No significant reactivity was observed with the human parvovirus B19 VP2 or influenza virus H5 hemaglutinin (H5) antiserum (Figure 2B). These results are consistent with those reported by Kantola et al [22].

### Screening of Control Human Serum Samples

**Figure 3:**
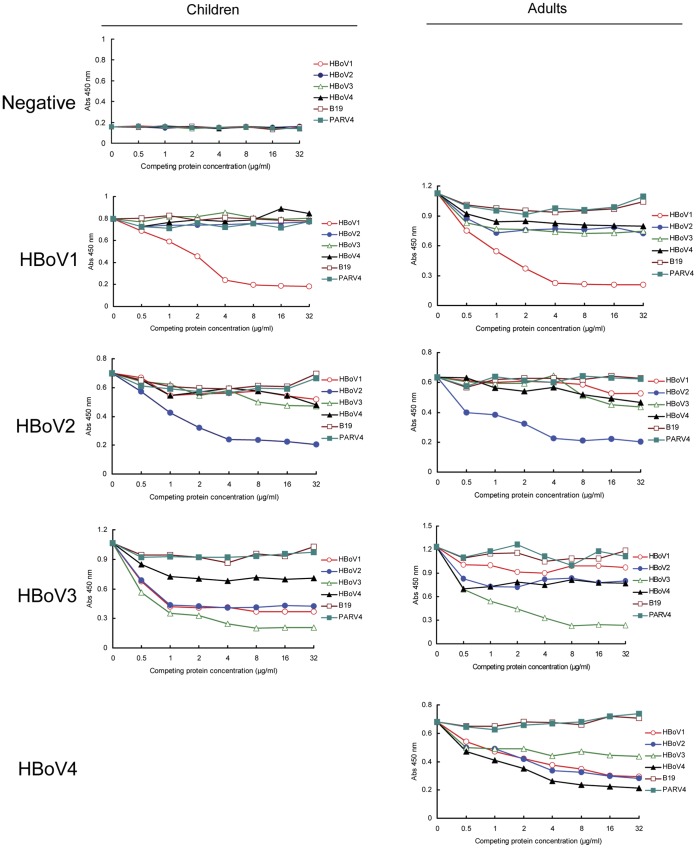
Reactivity of human serum IgG with HBoV VP2 virus-like particles after competition with soluble heterologous HBoV VLPs. VLP concentrations are shown on the x-axis. Sera from three children and four adults were positive for HBoV1, 2, 3, or 4. The negative sera samples that were from infants aged 0-6 months, were identified using Western blot analysis. Human parvovirus B19 and PARV4 VP2 proteins were used as controls.

To develop ELISA assays that can detect antibodies against HBoV species, we first sought to identify HBoV-positive and -negative sera samples using Western blot analysis. To obtain negative sera samples, we tested the reactivity of sera samples collected from infants aged 0-6 months who visited Beijing Children’s Hospital for regular health check-ups against HBoV VLPs. Only the samples that were negative for HBoV1, 2, 3, and 4 VLPs simultaneously were selected as the negative samples. The negativity of those samples was confirmed using a competition ELISA (cELISA) assay, in which the absorbance value at 450 nm did not change significantly with or without competition assays (Figure 3). Serum samples identified by cELISA as positive for individual HBoV species were obtained from 3 children and 4 adults and used as positive controls. However, we did not obtain a serum sample that was positive only for HBoV4 based on the assay; we used a sample from an adult that was IgG positive for HBoV1, HBoV2 and HBoV4. To determine the concentration of VLPs required for exhaustive antibody competition of HBoV1-4, the serum samples were monocompeted with concentrations of HBoV1, 2, 3, or 4 VLPs ranging from 0-32 µg/mL (Figure 3). We found that HBoV VLPs concentration of 16 µg/mL was the effective concentration to perform the VLP-based competition assays.

### Development of ELISA Methods for Detecting Anti-HBoV IgG Antibodies

To determine the seroprevalence of HBoV1, 2, 3, and 4 in humans, we developed an ELISA protocol for detecting IgG antibodies against HBoV1-4 using VLPs as coating antigens. The concentration of the coated VLPs (0.125 µg/mL) for this ELISA assay was optimized using chessboard titration tests. The positive sera and negative sera showed different results at 1∶200 serum dilutions. As the results obtained using lower serum dilutions were similar to those obtained at 1∶200 (data not shown), the 1∶200 dilution was used in subsequent ELISA analysis of serum samples. To determine the cut-off values for the ELISA, we determined the mean values and standard deviation of negative sera for HBoV1, 2, 3, and 4 using HBoV VLP ELISA at dilutions of 1∶200. We used the mean absorbance at 450 nm of the negative sera plus three-folds the standard deviation as the cut-off values, as previously described [19], [31]. For HBoV1, 2, 3 and 4, the cut-off values were 0.344, 0.304, 0.321, and 0.31, respectively. A sample was considered positive for HBoV1, 2, 3, or 4 if its absorbance at 450 nm was above the cut-off value of the respective species in ELISA.

As there are cross-reactivities between HBoV species in ELISA assay, we developed a cELISA assay to evaluate the seroprevalence of each HBoV species. We evaluated the specificity of this protocol using heterologous competition assays with parvovirus B19 and human parvovirus 4 (PARV4) VP2. Neither human parvovirus B19 nor PARV4 VP2 inhibited the reactivity of IgG against HBoV1, 2, 3, or 4 in the serum from adults or children (Figure 3). These results suggest that there is no antigenic cross-reactivity between HBoVs VP2 and human parvovirus B19 VP2 or between HBoVs VP2 and PARV4 VP2. To confirm the specificity of this ELISA protocol, we tested sera from 15 adults that were positive for human parvovirus B19. In the HBoV VLP ELISA, we did not detect false-positive signals for HBoVs (data not shown).

### Seroprevalence of HBoVs in Adults

**Figure 4:**
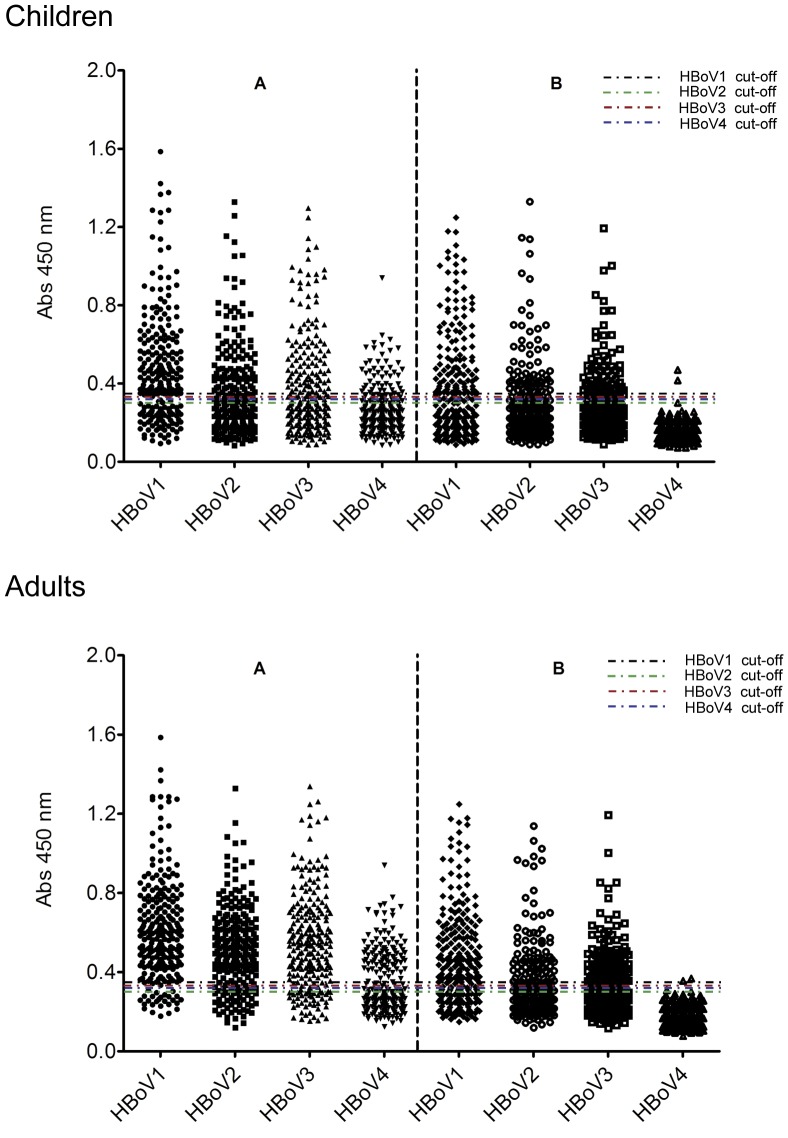
IgG reactivity of 244 sera from healthy children and of 142 sera from healthy adults with VLPs of HBoV1-4 (indicated on the x axis). The sera were not competed (A), or competed with heterologous VLPs of the other three HBoVs (B) prior to ELISA assays. The net reactivity was calculated as the raw absorbance value subtracted with the corresponding residual absorbance value from homologous competition. The mean of serum absorbance value are marked with red line. The IgG cut-off values of HBoV1 (0.344), HBoV2 (0.304), HBoV3 (0.321) and HBoV4 (0.31) are shown for reference.

**Figure 5:**
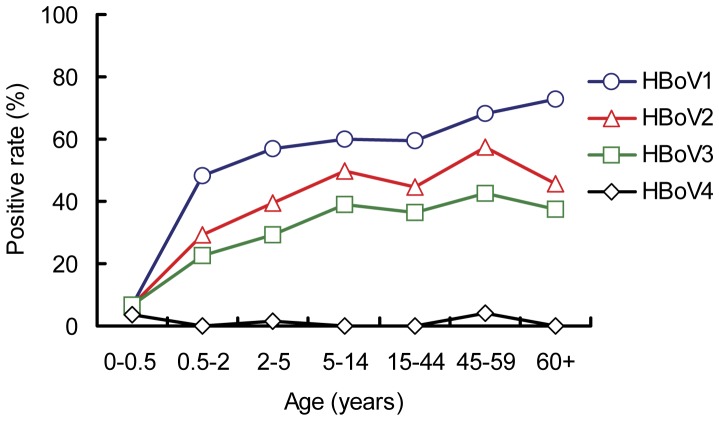
Seropositive rates of IgG antibodies against HBoV species in different age groups. IgG antibodies against HBoV1, 2, 3, and 4 were detected by competition ELISA at a dilution of 1∶200. All serum samples were grouped based on age, as indicated by the x-axis labels.

To determine the seroprevalence of HBoV1-4 in adults, we used the HBoV VLPs ELISA method to detect IgG antibodies against HBoV1-4 in 142 serum samples collected from healthy individuals 15-70 years old. Without competition, more than 90% samples were positive for HBoV1, HBoV2, and HBoV3; 73 (51.4%) samples were positive for HBoV4 (Table 1). However, these IgG seroprevalences decreased with competition by VLPs of heterologous HBoV species (Figure 4). The cELISA resulted in seroprevalences of 66.9%, 49.3%, 38.7%, and 1.4%, for HBoV1, 2, 3, and 4 IgG, respectively. The seropositive rate of HBoV1 was higher than that of HBoV2, HBoV3, and HBoV4 (χ^2^ = 23,*P*<0.01) (Table 1 and Figure 5).

**Table 1:** Immunoglobulin G (IgG) seroprevalences of 4 human bocaviruses among healthy individuals without/with antigen competition.

Age (years)	HBoV1,	HBoV2	HBoV3	HBoV4
All chidren (n = 244)	166^a^/122^b^ (68^c^/50^d^)	119/90 (48.8/36.9)	121/70 (49.6/28.7)	55/2 (22.5/0.8)
Children by age				
0–0.5 (n = 29)	8/2 (27.6/6.9)	3/2 (10.3/6.9)	5/2 (17.2/6.9)	3/1 (10.3/3.4)
0.5–2 (n = 62)	4/30 (67.8/48.4)	25/18 (40.3/29)	27/14 (43.5/22.6)	10/0 (16.1/0)
2–5 (n = 58)	42/33 (72.4/56.9)	33/23(56.9/39.7)	33/17 (56.9/29.3)	15/1 (25.9/1.7)
5–14 (n = 95)	74/57 (77.9/60)	58/47 (61.1/49.5)	56/37 (58.9/38.9)	30/0 (31.6/0)
All adults (n = 142)	141/95 (99.3/66.9)	137/70 (96.5/49.3)	137/55 (96.5/38.7)	73/2 (51.4/1.4)
Adults by age				
15–44 (n = 47)	46/28 (97.9/59.6)	45/21 (95.7/44.7)	46/17 (97.9/36.2)	25/0 (53.2/0)
45–59 (n = 47)	47/32 (100/68.1)	47/27 (100/57.4)	47/20 (100/42.6)	31/2 (66/4.3)
≥60 (n = 48)	48/35 (100/72.9)	45/22 (93.8/45.8)	44/18 (91.7/37.5)	17/0 (35.4/0)

a Number of positive samples without antigen competition.

b Number of positive samples with antigen competition.

c Percentage of positive samples without antigen competition.

d Percentage of positive samples with antigen competition.

Twenty-five of the 142 (17.6%) adult samples that were positive for IgG antibody against HBoV1, 2, and 3, and 17 (12%) that were positive for HBoV4 in the ELISA were negative for IgG against all 4 HBoVs in the cELISA. Among positive serum samples against single HBoV species, there were 26 for HBoV1, eight for HBoV2, and two for HBoV3 based on the cELISA results.

### Seroprevalence of HBoVs in Children

The seropositive rates of HBoV1, 2, and 3 in children increased with age according to results from the ELISA without competition. The rates for HBoV1, 2, and 3 reached to 77.9%, 61.1%, and 58.9%, respectively, in the age group 5-14 years. The seroprevalence for HBoV4 among children aged 0-0.5 year and 5-14 years increased from 10.3% to 31.6% (Table 1). However, according to results of the cELISA, the seroprevalences of HBoV1, 2, 3, and 4 IgG decreased about 10-27% (Table 1, Figure 5). The seroprevalence among children aged 0-0.5 years and children aged 5-14 years increased from 6.9% to 60%, 6.9% to 49.5%, and 6.9% to 38.9% for HBoV1, HBoV2, and HBoV3, respectively, in the cELISA.

Of note, among 29 infants aged 0-6 months, two (6.9%) were positive for HBoV1, 2, and 3 IgG, and one (3.4%) was positive for HBoV4. The detection of HBoVs IgG antibodies among children aged 0-6 months may be due to the presence of maternal antibodies. However, the seropositive rate of HBoV1 was higher than that of HBoV2, 3, or 4 in the age groups of 0.5-2 years, 2-5 years, and 5-14 years (χ^2^ = 10.1,*P*<0.01; χ^2^ = 9.3, *P*<0.01; χ^2^ = 8.42,*P*<0.05, respectively). Based on the results of the cELISA, there were 38 (15.6%) individuals positive for HBoV1, nine (3.7%) for HBoV2, and two (0.8%) for HBoV3 among positive serum samples against single HBoV species. Notably, the two samples positive for HBoV4 IgG were also positive for HBoV1, 2, and 3 IgG.

### Seroprevalence and Seroconversion Among Children with Acute HBoV1 Infection

To characterize the antibody response after HBoV infection further, we measured the IgG antibody in 31 pairs of sera samples that were collected from children with ARTIs. All these children were positive for HBoV1 according to PCR analysis. Based on cELISA, the seroprevalences of HBoV1, 2, 3, and 4 in acute-phase sera were 29%, 25.8%, 16.1%, and 0%, respectively. Of the 31 samples, ten (32.3%) showed an IgG seroconversion for HBoV1, eight (25.8%) for HBoV2, two (6.5%) for HBoV3, and none for HBoV4 (Table2). Two pairs of sera showed concurrent seroconversions to HBoV1 and HBoV2, and one pair showed concurrent seroconversions to HBoV1, HBoV2, and HBoV3. In addition, absorbance at 450 nm increasing in the convalescent-phase serum was observed in seven children (22.6%) who were positive for HBoV1 IgG and eight (25.8%) children who were positive for HBoV2 IgG positive at the acute-phase.

**Table 2:** HBoV1-4 IgG seroconversion in 31 paired sera samples that were collected from HBoV1-positive children with acute respiratory tract infections.

HBoV species	Acute-phase serum	Convalescent-phase serum	Seroconversion
HBoV1	9^a^ (29)^b^	19 (61.3)	10 (32.3)
HBoV2	8 (25.8)	16 (51.6)	8 (25.8)
HBoV3	5 (16.1)	7 (22.6)	2 (6.5)
HBoV4	0 (0)	0 (0)	0 (0)

a .Number of positive samples.

b .Percentage of positive samples.

## Discussion

In this study, we evaluated the cross-reactivity of mouse antisera against HBoV1, 2, 3, and 4 VP2 with VP2 VLPs for four species of human bocaviruses. Considerable cross-reactivity was found among the four HBoV species. However, human IgG antibodies against HBoV did not cross react with human parvovirus B19 and PARV4. These findings are consistent with those of a previous report [22], suggesting that to obtain accurate data of the seroprevalences of the different HBoV species, it is necessary to correct for cross-reactivity between the four HBoVs. Comparison of the VP2 protein sequences of HBoV1-4 revealed a high degree of similarity. These similarities may account for the cross-reactivity of these viruses, and should be confirmed through epitope analysis. Human parvovirus B19 and PARV4 have no antigen cross-reactivity with HBoVs, suggesting that human parvovirus B19 and PARV4 antibodies do not interfere with the HBoV seroprevalence results. The specificity of human bocavirus VLP-based ELISA with human sera has also been demonstrated with human parvovirus B19 VP2 and PARV 4 VP2 by Kantola et al [22].

We used a VLP-based ELISA to assess the seroprevalence of HBoV1-4 in healthy individuals in Beijing, China, ranging from 0 to 70 years old. Our results show that the seroprevalences of HBoV1, 2, and 3 range from 40.3-67.8% in children 0.5-2 years old and are up to 100% in adults. To eliminate the interference of antibody cross-reactivity, the results were corrected with a cELISA. The results of our cELISA suggest that the seroprevalences of HBoVs are significantly lower than those obtained without competition, especially in adults. These findings further indicate a high degree of antigenic cross-reactivity between HBoV1-4. Our findings are consistent with those of a recent study of Finish and Pakistani individuals, in which the seroprevalences of HBoVs decreased after depletion of heterologous HBoV reactive antibodies [22].

Our results from the cELISA are lower than those previously reported for HBoV1 in adults and children [18], [19], [32], where HBoV1 antibodies against HBoV1 VP2 were detected by ELISA. Hence, the seroprevalence of HBoVs may be overestimated due to the serological cross-reactivity among the four HBoV species in previous studies. However, according to our cELISA results, the seroprevalence of HBoV1-3 in Beijing adults is somewhat higher than that reported in Finnish and Pakistani adults [22]. This disparity may be attributed to the difference of geographical location [22].

Our age stratification data indicates that HBoVs circulate widely in the human population, and the primary infection with HBoV1-3 occurs in children aged six months and older after the maternal antibodies have waned. The seropositive rates of IgG antibodies against HBoV2, 3 and 4 were lower than that of HBoV1 in individuals of ≥0.5 years old. These data indicate that HBoV1 is the predominant circulating species of HBoV in Beijing, whereas HBoV2-4 do not seem to have a major impact on HBoV infections. This finding agrees with reports on HBoVs prevalence obtained from most parts of the world using DNA analysis [10], [14], [16]. Of note, our finding that the seroprevalence of HBoV4 is much lower than that of HBoV1-3 is consistent with the rare detection of HBoV4 DNA in clinical samples [9]. Overall, the seroprevalence of HBoVs indicated by results of the cELISA decreased less in children than in adults in comparison to the results without competition, indicating that HBoV infections are more specific in children, especially for HBoV1 [22]. These results suggest that HBoV species may play differential roles in disease.

Our results show that 17.6% of the adults who were positive for IgG against HBoV1, 2, 3, or 4 based on the ELISA results were negative for IgG against all four HBoVs based on the cELISA results. This finding may be due to antibody waning in the individuals with low IgG levels [22] or to low-affinity antibodies [20], [33].

To assess the antibody response of HBoV1-4 IgG in children with acute respiratory tract infections, we used cELISA to determine the seroconversion rate of 31 paired sera from children who were positive for HBoV1 according to PCR analysis. We found that the HBoV1-specific seroprevalence was higher in convalescent sera than in acute sera. However, heterotypic seroconversion against HBoV2 and HBoV3 was also observed. Moreover, for some patients who were positive for HBoV1 and HBoV2 IgG at the acute phase, the absorbance value was higher in convalescent sera. These data may suggest the concurrent production of antibodies against HBoV2 and 3 during the infection of HBoV1, as it has been shown that B-cell memory can be boosted either by the homologous virus or by heterologous, yet immunological related virus [20], [34], [35].

Overall, our findings suggest a differential prevalence of HBoV species in healthy individuals aged 0-70 years old. HBoV1 appears to be the dominant species responsible for HBoV infections among HBoV1, 2, 3, and 4 in Beijing, China. The seroprevalence of HBoV1-3 increased with age in children. Our study provides a basis for future evaluation of the epidemiology, genotype distribution, and pathogenesis of HBoVs worldwide.

## Materials and Methods

### Serum Specimens

Serum specimens were collected from 386 healthy individuals aged 0 to 70 years in 2008; 244 specimens were from infants and children who visited Beijing Children’s Hospital for regular health check-ups. The 142 specimens from adults were provided by the Beijing Blood Center. Exclusion criteria for all subjects included pregnancy, any abnormalities in renal and liver function tests, HIV/AIDS, sexual transmitted diseases, tumor, recurrent or acute infection, and medication. None of the subjects had any respiratory infection for at least three months prior to the blood samples being taken. In addition, paired acute-phase (at the time of admission) and convalescent -phase (2 weeks after disease onset) serum samples were collected from 31 children (median age 17 months; range of 1 month to 9 years) with acute lower respiratory tract infections (ALRTIs) when they were hospitalized at the Beijing Children’s Hospital. The DNA of HBoV1, but not HBoV2-4, was detected in the nasopharyngeal aspirates of these 31 patients at the time of admission by nested PCR and sequence analysis using primers targeting the viral proteins (VP) 1/2 region [9]. All serum samples were stored at −80°C prior to use.

Written informed consent was obtained from all participants or guardians on behalf of children. This study was approved by the ethical review committee of the Institute of Pathogen Biology, Chinese Academy of Medical Sciences.

### Phylogenetic Analysis

The full-length VP2 genes of HBoV1-4 were used for virus-like particle (VLP) production in this study. HBoV1-3 VP2 genes were amplified from HBoV-positive stool specimens (111-BJ07, 211-BJ07, and 46-BJ07; GenBank accession numbers: JQ240469, JQ240470 and HM132056) [15]. HBoV1 is 1,629 bp (nt 3,373-5,001 according to HBoV1 strain ST1, GenBank accession number DQ000495), HBoV2 is 1,617 bp (nt 3,306-4,922 according to HBoV2 strain PK2255, GenBank accession number FJ170279), and HBoV3 is 1,620 bp (nt 3,410-5,029 according to HBoV3 strain W471, GenBank accession number EU918736) in length. The VP2 genes of HBoV4 (1626 bp in length, nt3331-4956 based on HBoV4 strain NI-385, GenBank accession number NC012729) were synthesized by Sangon Biotech (Shanghai, China). These genes were verified by phylogenetic analysis using the Clustal W and MegAlign programs in the MEGA 4.0 software package [36]. The phylogenetic tree with 1,000 bootstrap replicates was generated based on the complete sequences of the VP2 genes used in this study and reference sequences from GenBank. HBoV1 strains ST1, ST2, and TW2888_06; HBoV2 strains W153, PK-2255, LZ55602, and 277-BJ07; HBoV3 strains W471 and W855; and HBoV4 strains NI-385 were used as reference sequences (GenBank accession numbers DQ000495, DQ000496, EU984237, EU082213, FJ170279, GU301645, JQ240471, EU918736, FJ948861, NC012729, respectively).

### Recombinant Protein Expression and Production of VLPs

The full-length VP2 genes of HBoV1, 2, 3, and 4 were cloned into the baculovirus expression vector pFastbac1 and expressed using Bac-to-Bac® Baculovirus Expression System (Invitrogen, Carlsbad, CA), according to the manufacturer’s protocol. VLPs were obtained as previously described [17], [37]. High Five cells (Invitrogen) were infected with recombinant baculoviruses at a multiplicity of infection (MOI) of five and harvested after three days. Cells were suspended in 25 mM NaHCO_3_ solution at 2×10^7^ cells/mL and kept on ice for 30 min. After centrifugation at 18,000 rpm for 10 min at 4°C, (NH4)_2_SO_4_ was added to the supernatants at a final concentration of 20% (w/v). Precipitants were harvested by centrifugation and dissolved in CsCl solutions with densities of 1.4 g/mL in Tris-EDTA buffer (10 mmol/L Tris, pH 8.7, 1 mmol/L EDTA, and 0.5% Triton X-100). After centrifugation at 35,000 rpm and 18°C for 39 h in a SW41 rotor centrifuge (Beckman Coulter, Fullerton, CA), the fractions were analyzed by SDS-PAGE and Western blot using mouse sera against HBoV VP2 proteins. A Tecnai12 transmission electron microscope (FEI, Hillsboro, OR) at 80 kV was used to verify the morphology of the VLPs. SDS-PAGE and Western blot analysis were used to identify the VLPs [18].

Recombinant VP2 proteins of HBoV1, 2, 3 and 4 were also expressed in *E. coli* Rosetta (DE3) (Novagen, Madison,WI,) cells and purified as previously described [37]. The genes used in prokaryotic cloning were the same as those used in a baculovirus expression system. The recombinant antigens were used to immunize mice to produce antibodies (see below).

As controls, human parvovirus B19 VP2 was expressed, as described previously [38], using the construct pFastBac1B19VP2 provided by Dr. Xiaohui Zou at the National Institute for Viral Disease Control and Prevention, Chinese Center for Disease control and Prevention. Additionally, the PARV4 VP2 gene (1,659 bp in length, nt 3,464-5,122 based on the NC_007018 reference sequence) was synthesized by Sangon Biotech (Shanghai, China) and cloned into baculovirus vector pFBGP67-His [39]. The recombinant baculoviruses were generated in Sf9 cells using the Bac-to-Bac® Baculovirus Expression System (Invitrogen) protocol provided by the manufacturer. High Five cells were infected with recombinant baculovirus expressing PARV4 VP2 gene at a MOI of five. The infected cells were collected three days post-infection and purified using a HisTrap HP 1 ml column (GE Healthcare, Waukesha, WI). The concentrations of all purified protein were determined using the Pierce BCA Protein Assay Kit (Thermo Scientific, Rockford, IL) and stored at −80°C prior to use.

### ELISA

ELISA was used to determine the anti-HBoV antibodies, as described elsewhere [40]. The purified HBoV VLPs were used as coating antigen (0.125 µg/mL). The absorbance of each serum sample was read at 450 nm and the mean values of the duplicate samples were calculated. Sera pooled from ten samples showing HBoV-specific IgG responses were used as the internal references in all experiments.

### Analysis of Cross-reactivity between HBoV Species

To minimize false positive results of the ELISA assay due to impurities in immunizing and coating antigens, the coating antigen and the protein used to prepare mouse antibodies were derived from a baculovirus expression system and a prokaryotic expression system. BALB/c mice were injected subcutaneously with the purified proteins obtained from *E. coli*. This study was carried out in accordance with the animal experiment regulations of the Chinese government. All animal experiments were performed in the facilities of the Institute of Laboratory Animal Sciences (ILAS), Chinese Academy of Medical Sciences (CAMS). All experimental procedures were approved (license number SCXKJ2009-0017) and supervised by the Animal Protection and Usage Committee of ILAS, CAMS. Sera collected from the treated mice were purified using protein-G sepharose columns (GE Healthcare, Waukesha, WI) and purified IgG antibody were quantified using Pierce BCA Protein Assay Kit (Thermo Scientific). The purified mouse IgG antibodies were serially diluted from 8 µg/mL to 0.008 µg/mL. Sera from pre-immune mice served as the negative control. Rabbit antiserum against human parvovirus B19 (a gift from Dr. Xia Xiao of National Institute for Viral Disease Control and Prevention, Chinese Center for Disease control and Prevention) and mice antiserum against influenza virus H5 hemaglutinin (HA) [39] were used as unrelated controls. The cross-reactivity between HBoV1, 2, 3, and 4 was tested using Western blot analysis and ELISA.

### VLP-based Competition ELISA Assays

To measure antibodies specific to VP2 antigen of an individual HBoV species (HBoV1, 2, 3, or 4), antibodies in serum samples were absorbed with heterologous VLPs of the other three HBoVs, as previously described [22]. Briefly, HBoV VLPs were serially diluted from 32 µg/mL to 0.5 µg/mL to determine the concentration needed for effective competition between cross-reactive antibodies. For detection of specific HBoV antibodies, three heterologous HBoV VLPs of 16 µg/mL were added to a 1∶200 dilution of plasma [17] and incubated for 2 hr at 4°C prior to performing the ELISA assay. In parallel with the heterologous competition, human sera samples were used to compete with VLP that was homologous to the immobilized antigen. The absorbance at 450 nm was residual absorbance value. Net absorbance values were calculated by subtracting the residual absorbance value from the raw absorbance value read at 450 nm [22].

### Statistical Analysis

Seropositive rates were evaluated using χ^2^ tests. A *P* value ≤0.05 was considered significant.
